# Reversible Elevation of Tryptase Over the Individual's Baseline: Why is It the Best Biomarker for Severe Systemic Mast Cell Activation and MCAS?

**DOI:** 10.1007/s11882-024-01124-2

**Published:** 2024-02-03

**Authors:** Peter Valent, Cem Akin, Michel Arock

**Affiliations:** 1 Department of Internal Medicine I, Division of Hematology and Hemostaseology, Medical University of Vienna, Vienna, Austria; 2 Ludwig Boltzmann Institute for Hematology and Oncology, Medical University of Vienna, Vienna, Austria; 3 Division of Allergy and Clinical Immunology, University of Michigan, Ann Arbor, MI USA; 4 Platform of Molecular Analysis for Mastocytosis and MCAD (CEREMAST), Department of Biological Hematology, Pitié-Salpêtrière Hospital, AP-HP, Paris Sorbonne University, Paris, France

**Keywords:** MCAS, Anaphylaxis, Allergy, Mast cells, Mastocytosis, Histamine, Tryptase

## Abstract

**Purpose of Review:**

Mast cell (MC) activation syndromes (MCAS) are conditions defined by recurrent episodes of severe systemic anaphylaxis or similar systemic events triggered by MC-derived mediators that can be measured in biological fluids. Since some symptoms of MC activation may occur due to other, non-MC etiologies and lead to confusion over diagnosis, it is of crucial importance to document the involvement of MC and their products in the patients´ symptomatology.

**Recent Findings:**

The most specific and generally accepted marker of severe systemic MC activation is an event-related, transient increase in the serum tryptase level over the individual baseline of the affected individual. However, baseline concentrations of serum tryptase vary among donors, depending on the genetic background, age, kidney function, and underlying disease. As a result, it is of critical importance to provide a flexible equation that defines the diagnostic increase in tryptase qualifying as MCAS criterion in all patients, all situations, and all ranges of baseline serum tryptase. In 2012, the consensus group proposed the 120% + 2 ng/ml formula, which covers the great majority of groups, including cases with low, normal, or elevated basal serum tryptase level.

**Summary:**

This formula has been validated in subsequent studies and has proven to be a robust and consistent diagnostic criterion of MCAS. The present article is discussing the impact of this formula and possible limitations as well as alternative markers and mediators that may be indicative of MCAS.

## Introduction

Mast cells (MC) are multi-functional leukocytes that reside in various organs and tissues and play an important role in diverse immunological processes and pathologies [1–3, 4•, 5•]. In common with other leukocytes, including tissue-resident macrophages, basophils, and other immune effector cells, MC derive from hematopoietic stem and progenitor cells. MC synthesize and release various pro-inflammatory mediators, including histamine, leukotrienes, and prostaglandins as well as specific proteases (tryptase, chymase) and distinct proteoglycans, including heparin, which is largely a MC-specific compound [1–3, 4•]. During an anaphylactic or allergic reaction caused by immunoglobulin-E receptor (IgER) cross-linking, MC instantly release their mediators into the extracellular space [1–3, 4•, 6–9]. Apart from allergens, a number of other triggers and receptor-activating ligands can provoke MC activation and can thus participate in hypersensitivity reactions [3, 6, 8, 9].

Tryptases are a group of serine proteases that are almost exclusively synthesized and stored in MC, and less abundantly in basophils [10, 11, 12•, 13]. In MC, tryptases have been described to account for up to 30% of their total protein content [12•]. Whereas the mature tryptase proteins are preferentially stored in the heparin-containing, secretory metachromatic granules of MC, the enzymatically inactive precursor (pro) forms of tryptase, are released continuously by resting cells, independent of the maturation stage of MC, the tissue and organ site, and the underlying pathology [10, 11, 12•, 14, 15]. As a result of steady-state release of the enzyme and its chemical stability under various conditions, the basal serum tryptase level in individual (healthy) donors is remarkably consistent, although slight variations (fluctuations) of the individual baseline have been described, especially in individuals with hereditary alpha tryptasemia (HαT), a condition leading to elevated baseline serum tryptase levels due to multiple copy numbers of TPSAB1 encoding alpha tryptase [16, 17, 18•]. The basal serum tryptase level can also be elevated in various reactive processes and in diverse malignancies but usually remains stable in individual patients when tested over time, unless the malignancy progresses or is successfully treated [19–22, 23•, 24].

However, in the course of a severe systemic MC activation (anaphylaxis) where large quantities of mediator substances, including tryptase, are released from MC, serum tryptase levels usually increase substantially over the individual´s baseline [23•, 24–27, 28•]. Thereafter, serum tryptase levels return to the individual´s baseline, a process that usually takes several hours, depending on reaction-type and response to therapy [23•, 24–27, 28•]. The sustained tryptase peak provides a diagnostic window of testing for several hours (recommended: up to 4 h) after the event occurred.

In the past 30 years, basal serum tryptase has emerged as a preferred biochemical marker of the total body burden of MC, whereas a substantial, event-related increase in tryptase over the individual´s baseline has emerged as the preferred biochemical marker of severe systemic MC activation and anaphylaxis [19–27, 28•, 29•, 30, 31•, 32, 33, 34•].

Other MC-derived compounds and/or their metabolites, such as histamine or prostaglandin D2 (PGD2), may also serve as markers of MC activation and anaphylaxis under certain conditions [3, 6–9, 27]. However, these molecules are less specific for MC and less specific for severe anaphylaxis, but are also found elevated in other (less severe) allergic reactions, and have not been studied extensively in non-allergic diseases. In addition, only a few studies have assessed the event-related, diagnostic, increase over the individual´s baseline of such other MC mediators in the context of MC activation and anaphylaxis [35].

Severe systemic MC activation resembling anaphylaxis is most commonly found in patients with IgE-dependent allergies [1–3, 4•, 6–9]. When the symptoms are severe and recurrent and involve at least two organ systems, a MC activation syndrome (MCAS) may be diagnosed, provided that MCAS criteria are fulfilled [29•, 30, 31•, 32, 33, 34•]. In these patients, MCAS is further divided into distinct MCAS variants, based on the underlying pathology. In particular, MCAS can be split into i) primary (mono/clonal) MCAS defined by the presence of *KIT*-mutated clonal MC, ii) secondary MCAS characterized by an underlying allergic or other reactive disorder triggering MC activation, and iii) idiopathic MCAS where neither clonal MC nor an underlying allergic or reactive disease triggering MC activation, can be identified [29•, 30, 31•, 32, 33, 34•].

In many patients with allergic disorders or other MC-related pathologies, signs and symptoms of MC activation are observed and are relevant clinically, but may be localized or not severe enough to diagnose MCAS [33, 34•]. Indeed, the criteria to diagnose MCAS are stringent and specific for severe systemic MC activation. According to the proposal of the EU/US consensus group, MCAS can be diagnosed when the following diagnostic criteria are met: i) recurrent episodic occurrence of typical systemic symptoms that are induced by MC mediators and are involving two or more organ-systems, ii) an event-related, transient, increase in the serum tryptase concentration to at least 120% of the individual´s baseline plus 2 ng/ml, and iii) a documented response to drugs directed against MC mediator effects (for example: histamine receptor blocker) or MC activation (MC stabilizers) [29•, 30, 31•, 32, 33, 34•].

In the present article, we discuss the clinical value and limitations of tryptase as a most specific and robust biochemical marker of severe systemic MC activation and MCAS. Moreover, we explain why the increase in tryptase over the individual´s baseline to at least 120% + 2 ng/mL is a specific qualifying criterion of MCAS.

## The Basal Serum Tryptase Level in Healthy Controls

The basal tryptase level is remarkably stable over time in individual healthy donors provided that no underlying disease associated with MC activation or MC expansion is present [16, 17, 18•]. Moreover, in contrast to many other MC-derived mediators (histamine, PGD2, heparin, others), tryptase is a biologically stable protein that is not degraded, removed, or de-activated by changes in temperature, by other plasma proteins, or by short-term storage.

However, the basal serum tryptase level varies among healthy individuals, depending on the genetic background, sex, age, kidney function, and presence of an underlying disease associated with MC expansion and/or activation [16, 17, 18•].

The normal serum tryptase level ranges between 0 and 15 ng/mL in healthy adults of Caucasian origin [18•, 19–21]. In those with HαT, tryptase levels are either within normal range or are elevated (> 15 ng/mL), depending on the number of extra-copies of the *TPSAB1* gene, and independent of the symptoms recorded or the presence of co-morbidities. When excluding HαT carriers from the pool of healthy controls, the normal basal serum tryptase level amounts to approximately 0–11.4 ng/mL [16, 17, 18•]. However, most HαT carriers are asymptomatic or have non-specific co-morbidities not attributable to MC activation or tryptase, so that these individuals must be included in calculating the normal range of serum tryptase levels [36]. Therefore, these individuals (at least asymptomatic) should be included when defining the normal range of basal serum tryptase [18•]. It is also worth noting that many individuals with HαT have tryptase levels < 10 ng/mL or even < 8 ng/mL [37].

## Etiologies Underlying an Elevated Basal Serum Tryptase Level

A number of conditions and pathologies can cause an elevated basal serum tryptase level. The most prevalent underlying condition is HαT, an autosomal dominant genetic trait associated with two or multiple copy numbers of the *TPSAB1* gene encoding alpha tryptase [38•, 39, 40•]. HαT is detectable in approximately 4–7.5% of the general population in the Western world [36, 41, 42•]. Many of these individuals have slightly or moderately increased tryptase levels [36, 38•, 39, 40•, 41, 42•]. The excess of tryptase also correlates with the total copy numbers of the *TPSAB1* gene. However, most of the HαT carriers have only one extra gene copy and many of these cases present with a normal serum tryptase level [37].

It is important to state that HαT per se cannot be regarded as a biomarker of anaphylaxis or MCAS. Rather, HαT appears to be a modifying factor predisposing for severe symptoms resulting from MC activation in patients who are suffering from an underlying allergic disease and/or a clonal MC disorder [36, 41, 42•]. Overall, the occurrence of severe symptoms of anaphylaxis cannot be predicted in individual patients by their *TPSAB1* gene status or their basal serum tryptase level.

Another condition that may produce elevated basal serum tryptase levels is a markedly reduced renal function in patients with chronic kidney disease [43, 44]. Tryptase levels may also increase during chronic infections associated with MC hyperplasia and in those who are treated with recombinant stem cell factor (= MC growth factor) [45].

Finally, basal tryptase levels may increase in patients with myeloid malignancies, including myelodysplastic syndromes (MDS), myeloproliferative neoplasm (MPN), chronic myeloid leukemia (CML), acute myeloid leukemia (AML), MDS/MPN overlap disorders, and mast cell neoplasms, including systemic mastocytosis (SM) (Table 1) [19–22, 40•, 42•]. Patients with eosinophil-related myeloid neoplasms with rearranged *PDGFR* fusion genes may also have increased basal serum tryptase levels (Table 1).

**Table 1:** Conditions associated with an elevated basal serum tryptase level

Condition	Typical range of serum tryptase in ng/ml*	Increased risk for MCAS
Hereditary alpha tryptasemia	15–50**	+/-***
Cutaneous mastocytosis	5–15	+
Indolent systemic mastocytosis	15–200	++
Smoldering systemic mastocytosis	200–1000	+
Advanced systemic mastocytosis****	100–1000	+
Myelodysplastic syndromes	10–50	-
Myeloproliferative neoplasms	10–100	-
Chronic eosinophilic leukemia	10–50	-
Myeloid neoplasms with *PDGFR* mutation	10–50	-
Chronic myeloid leukemia	10–50	-
Acute myeloid leukemia (AML)	10–1000	-
Core binding factor (CBF) AML	10–1000	-
Non-CBF AML	10–200	-
Chronic helminth infection	10–20	-***
Chronic renal failure	10–30	-

Table 1 was reproduced in slightly modified form from Valent et al., Int Arch Allergy Immunol [48] with the Editor´s permission

Score: ++ substantial risk to develop MCAS events one or more times per year despite therapy with anti-mediator-type drugs, + increased risk to develop MCAS events one or more times over several years, +/- MCAS events have been reported in individual patients, but the precise incidence is not clear, - no increased risk to develop MCAS events compared to the general population (± allergic individuals).

*Range of basal serum tryptase concentration where a majority of cases are found; **In patients with hereditary alpha tryptasemia, the serum tryptase level increases 7–15 ng/ml with each additional *TPSAB1* gene copy; in those with multiple copies, tryptase levels can increase up to 100 ng/ml; ***In these conditions, the incidence of severe anaphylaxis (MCAS) is not known although some reports suggest an increased risk; ****Advanced systemic mastocytosis includes patients with aggressive systemic mastocytosis and mast cell leukemia. In these patients, the basal tryptase level may increase to > 500 or even > 1000 ng/ml. Abbreviations: MCAS, mast cell activation syndrome

The highest levels of basal serum tryptase are found in patients with smoldering SM (SSM) and advanced SM, including MC leukemia (MCL) and some patients with AML, especially those who have core binding factor AML [19, 21, 46•, 47]. In these patients, basal serum tryptase levels may increase to over 1000 ng/mL (Table 1). In most patients with non-advanced SM, in most with HαT with more than 2 extra *TPSAB1* gene copy numbers, and some patients with high risk (accelerated phase) CML, basal serum tryptase levels are between 50 and 200 ng/mL. In all other groups of patients, basal serum tryptase levels are usually below 50 ng/mL. All in all, serum tryptase levels greatly vary among patients and among healthy controls, depending on genetic factors, renal function, and co-morbidities.

## Diagnostic Increase in Serum Tryptase in Anaphylaxis and MCAS: Scientific Basis and Discussion of the 120% + 2 ng/mL Formula

**Fig. 1:**
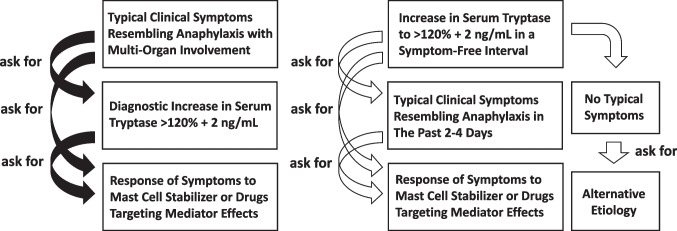
Step-wise approach and application of diagnostic criteria in patients with suspected mast cell activation syndrome (MCAS). In patients with clinical signs and symptoms of anaphylaxis (severe hypotension, collapse, acute urticaria, allergic asthma, red skin rash, edema, abdominal pain plus vomiting and diarrhea, others), it is a reasonable diagnostic approach to ask for an event-related increase in tryptase. To define the increase in tryptase, serum samples have to be collected during or shortly after the anaphylactic event and in a symptom-free interval. A diagnostic increase in the event-related tryptase > 120% + 2 ng/mL confirms the involvement of the mast cell (MC) lineage and serves as diagnostic criterion of MCAS. In these cases, the symptoms will respond to drugs stabilizing MC and/or drugs targeting MC mediator effects. In patients without clinical symptoms of MC activation or anaphylaxis, the tryptase level may also be increased to 120% + 2 ng/mL compared to previous examinations (right panel). In these cases, the etiology of the (further) increase in tryptase (for example: progression of mastocytosis) should be explored. If no such etiology is detectable, the increase may be due to a recent anaphylactic event (then the final diagnosis may be MCAS), or may be due to fluctuations in the basal serum tryptase: in these patients, the increase to > 120% + 2 ng/mL (over baseline) does not count as criterion of MCAS

During an anaphylactic reaction, MC activation (degranulation) and the massive (additional) release of tryptase usually leads to a substantial and transient increase in serum tryptase levels over the individual´s baseline (Fig. 1) [23•, 24–27, 28•]. In many cases, pre-event serum is not available and was not measured for basal serum tryptase levels. However, basal serum tryptase levels can also be determined in an event-free interval following anaphylaxis. In fact, after a short latency period of several hours following an anaphylactic event, tryptase levels return back to baseline. Therefore, it is important to follow the patients, and to measure the basal serum tryptase level after an event, preferably 24–48 h after resolution of all symptoms of an anaphylactic reaction. However, baseline levels of tryptase may also show some fluctuations over time in symptom-free intervals [16, 17, 18•].

In order to propose a robust minimal diagnostic increase in serum tryptase over the individual´s baseline indicative of severe systemic MC activation in all patients (those with very high levels, high baseline levels, and very low levels), a consensus equation was prepared during the 2010 working conference on MC disorders [31•]. This equation was also adjusted to slight variations in the individual´s baseline serum tryptase level and was created to exclude mild systemic or local forms of MC activation [31•, 48].

It is also worth noting that a persistently elevated serum tryptase level that may be found in patients with HαT or SM, is not indicative of severe systemic MC activation, but is indicative of increased basal secretion of tryptase in HαT, and an increased total body burden of MC in SM.

Overall, the idea was to develop a criterion that confirms with certainty that MC are involved in the reaction, and that the reaction is systemic and severe and caused by MC-derived mediator products. After a workgroup review of tryptase levels at baseline and after anaphylactic reactions, the resulting equation that was selected as a diagnostic criterion of MCAS appeared to be the 120% + 2 ng/mL formula [31•, 48]. This formula defines the minimal increase in serum tryptase that qualifies as robust sign of severe MC activation in individual donors and thus as criterion of MCAS [31•, 48]. The 120% + 2 ng/mL formula was initially tested against data published in the available literature and retrospective case report series. Later, the equation did undergo clinical validation by various independent study groups.

## Validation of the 120% + 2 ng/mL Equation in Clinical Practice

In the past 5 years, the value of the 120% + 2 ng/mL formula has been confirmed in several validation studies in patients with hypersensitivity reactions, defined allergies, and MC neoplasms. In patients with SM, the 120% + 2 ng/mL equation is a reliable marker of severe MC activation, that delineates between an anaphylactic (MCAS) event and less severe forms of MC activation (or other pathologies) not fulfilling MCAS criteria, independent of the variant of SM, co-morbidities, or basal tryptase concentrations [48–52]. These data confirmed previous studies that have shown that a substantial increase in tryptase is a reliable parameter to document MC activation during anaphylactic episodes in patients with SM [23•, 24, 26]. It has also been described that the 120% + 2 ng/mL formula is a robust equation to confirm perioperative anaphylaxis and anaphylaxis in children presenting to the emergency department [53•, 54, 55]. By contrast, in patients with local MC activation or less severe mediator-induce symptoms, including cases with pollen or food allergies, the serum tryptase level may remain below the 120% + 2 ng/mL threshold, even if the symptoms are clinically relevant [53•, 54–58]. It is also worth noting that the serum tryptase level may rarely exceed the individual´s baseline by 120% + 2 ng/mL in the absence of any signs or symptoms of MC activation or anaphylaxis [52] which may be due to natural variations (fluctuations) of the enzyme level, unrecognized MC activation (pre-analytical issues), or unrecognized kidney failure (Fig. 1). However, in this regard, it is of utmost importance to be aware of the practical algorithm that guides the clinician in the evaluation of patients with suspected MCAS (Fig. 1). In fact, this algorithm should be started by assessing the patient for signs and symptoms of anaphylaxis, and if such clinical signs (criteria) of MC activation are found, tryptase levels are measured during or shortly after the event as well as in a symptom-free interval (baseline level) (Fig. 1). However, MCAS criteria should not be applied in patients without symptoms, apparently mild symptoms, or symptoms that are not directly attributable to or less specific for MC activation. On the other hand, an increase in (basal) tryptase over the previous baseline in a routine test (symptom-free interval) should prompt the clinician to ask for potential causes, such as progression of an underlying disease (SM), decrease in kidney function, or recent anaphylaxis (Fig. 1). If indeed recent anaphylaxis is reported, MCAS criteria will (again) apply.

It is also important to state that severe systemic MC activation and symptoms of anaphylaxis (with or without fulfilled MCAS criteria) always have therapeutic implications. In fact, these patients usually need immediate therapy with anti-histamines, glucocorticosteroids and/or even epinephrine to bring the acute event under control. Moreover, depending on the underlying condition, these patients are often treated with MC stabilizers, KIT-targeting drugs, anti-IgE-based drugs or specific immunotherapy for long-term prophylaxis. In most cases, treatment of the acute event should (must) start in the absence of knowledge on basal serum tryptase levels and event-related tryptase.

However, it is important to collect these important laboratory test results for several reasons. First, an elevated basal serum tryptase level may be indicative of an underlying HαT, underlying SM, or other myeloid neoplasm. Indeed, in patients with severe IgE-dependent allergy and anaphylaxis, SM may be diagnosed, especially when a hymenoptera venom allergy is detected [58–61]. Second, a diagnostic increase in the event-related tryptase beyond 120% + 2 ng/mL of baseline will confirm the presence of MCAS and thus the involvement of the MC lineage.

## Alternative Biochemical Markers of MC Activation and MCAS

In the past 3 decades, a number of efforts have been made to define additional robust markers of severe MC activation and anaphylaxis. Most of these studies focused on other MC-derived chemical substances, such as histamine and its metabolites, prostaglandin D2 (PGD2) and its metabolites, or heparin [27, 62–67] (Table 2). The metabolites of histamine, PGD2 and LTC4 are often measured in urinary samples, since the primary mediators are unstable in serum and are rapidly metabolized. However, unlike heparin, these compounds are not specific for MC but also expressed in several other cell types (Table 2). For example, histamine is also displayed by basophils, and less abundantly by platelets, and may be spuriously ´elevated´ due to blood draw, storage issues, and/or processing conditions.

**Table 2:** Biomarkers indicating systemic severe mast cell activation in patients

Biomarker	Specificity for	Sensitivity in	Commonly used in
	mast cells	anaphylaxis	daily practice
Tryptase	+ +*	+**	++
Plasma histamine	+/-	+	+
Urinary histamine metabolites***	+/-	++	++
Prostaglandin D2 metabolites****	+/-	++	+
Urinary cysLT levels	-/+	++	+/-
Heparin	+++	-/+*****	-

Table 2 was reproduced with slight modifications from Valent et al., Int Arch Allergy Immunol [48] with the Editor´s permission

*Basophils express very low amounts of tryptase – but mast cells are a primary and major source of the enzyme; **The relatively low sensitivity of tryptase qualifies as a biomarker of massive mast cell activation and thus as a criterion of MCAS; ***Relevant 24-h urinary histamine metabolites include N-methylhistamine and N-methylimidazoleacetic acid; ****Among prostaglandin D2 metabolites the most commonly measured substance is urinary 11β-prostaglandinF2α; *****An increase in heparin is usually not measurable during an anaphylactic episode, unless the burden of mast cells is very high (like in mast cell leukemia). Abbreviations: cysLT, cysteinyl leukotriene

Another major problem is that no (minimal) event-related increase of these markers over the individual´s baseline documenting MC activation and anaphylaxis (and thus MCAS) has been defined so far. In addition, commercial assays to measure these mediators (and their metabolites) are not widely available in all centers. Furthermore, some of these molecules (urinary metabolites) have to be quantified in 24-h urine samples collected under certain guidelines, including dietary restrictions, to obtain reproducible results [62–66].

Finally, histamine and PGD2 metabolites also increase in various (MC-independent) reactive conditions and also in situations where only mild, but not severe MC activation is found, such as aspirin exacerbated respiratory disease and chronic urticaria. Therefore, these chemical compounds may better qualify as sensitive screen markers (or follow up markers) of MC activation, but may not qualify as specific markers or criteria of severe systemic MC activation and MCAS. The minimal threshold-increase of these compounds that would qualify as a robust sensitive marker of MC activation remains to be defined in clinical studies. Table 2 shows an overview of MC-dependent compounds, their specificity for MC, and their sensitivity in MC activation-related events and anaphylaxis.

## Concluding Remarks and Future Perspectives

Depending on the genetic background, underlying disease, and co-morbidities, baseline levels of serum tryptase greatly vary among individual donors. The most prevalent genetic condition associated with elevated tryptase is HαT, and the most prevalent MC neoplasm with elevated tryptase is SM. However, even in the groups of HαT and SM, tryptase level may be very low, normal, elevated or highly elevated. Therefore, a robust biomarker of MC activation needs flexibility to cover all ranges of basal tryptase. The 120% + 2 equation (also known as + 20% + 2 equation) fulfills this important criterion. In addition, a tryptase elevation to 120% + 2 over the individual´s baseline is specific for severe systemic MC activation (anaphylaxis) and thus MCAS, as in most individuals with local or less severe forms of MC activation, the 120% + 2 threshold is not reached. For these patients with less severe forms of anaphylaxis, more sensitive biomarkers of MC activation have to be applied. For example, an event-related increase in histamine, histamine metabolites or PGD2 metabolites may be indicative of MC activation. Whether these biomarkers are robust and specific enough to be employed as criteria of MC activation in patients remains to be determined in clinical trials and controlled validation studies.

## Data Availability

No datasets were generated or analysed during the current study.
